# Estimating the additional costs of living with a disability in the United Kingdom between 2013 and 2016

**DOI:** 10.1007/s10198-021-01366-1

**Published:** 2021-08-23

**Authors:** Lukas Schuelke, Luke Munford, Marcello Morciano

**Affiliations:** 1 Barcelona Graduate School of Economics, Barcelona, Spain; 2 School of Health Sciences, University of Manchester, Manchester, UK

**Keywords:** Disability, Standard of living, Equivalisation, Poverty, Welfare state, Extra costs, I14, I32, I38

## Abstract

In the United Kingdom, more than 20% of the population live with a disability. Past evidence shows that being disabled is associated with functional limitations that often cause social exclusion and poverty. Therefore, it is necessary to analyse the connection between disability and poverty. This paper examines whether households with disabled members face extra costs of living to attain the same standard of living as their peers without disabled members. The modelling framework is based on the standard of living approach which estimates the extra income required to close the gap between households with and without disabled members. We apply an ordered logit regression to data from the Family Resources Survey between 2013 and 2016 to analyse the relationship between standard of living, income, and disability, conditional on other explanatory variables. We find that households with disabled members face considerable extra costs that go beyond the transfer payment of the government. The average household with disabled members saw their weekly extra costs continually increase from £293 in 2013 to £326 in 2016 [2020 prices]. Therefore, the government needs to adjust welfare policies to address the problem of extra costs faced by households with disabled members.

## Introduction

In the United Kingdom (UK) more than one in five people are living with a disability, and the number continues to grow year on year [1]. To offer some perspective, roughly 13.9 million people are currently classified as disabled in the UK, which is more than the total population of the state of Pennsylvania, the fifth biggest state in the United States. Disability affects almost everyone—either directly by being personally disabled or indirectly through caring for family members or interacting with disabled persons at work or elsewhere. While for some, “*disability need not be an obstacle to success*”, for most having a disability comes with many limitations [2]. It is the imperative task of society and the government to remove barriers and promote the well-being and social inclusion of everyone in society [3]. In the UK, a welfare system is in place to promote an equal standard of living for people with disabilities. However, despite financial support from the government to protect vulnerable people, the risk of experiencing poverty and social exclusion as a disabled person is significantly higher than that of people without a disability [4]. Therefore, it is necessary to analyse the connection between disability and social exclusion, to address the impact of disability, and to prevent risks of exclusion that arise from poverty for disabled people. The underlying hypothesis is that a household with a disabled member (henceforth a ‘disabled household’), in diverting resources to goods and services which are required because of disability, experiences a lower standard of living than their non-disabled counterparts. A disabled household is defined as a household with at least one disabled person living there. The economic disparity arises from expenditure on disability-related goods and services and other opportunity costs. For example, disabled households may have to forego needed goods, because they need to divert income to cope with disability-related costs which in turn prevents them from participating more fully in their community. This paper aims to offer an estimate of the extra income necessary to close the gap in the United Kingdom—this is often called the “extra cost of disability” [5]. The paper will also assess the presence of time trends in the extra costs over the period from 2013 to 2016 in the UK. Results aim to inform on policy performance and formulation and promote evidence-based decision-making for public programmes of support for disabled people.

The Equality Act 2010 is the central legislative framework in the UK that governs equal opportunities in the wider society and safeguard disabled people. There are nine ‘protected characteristics’ and one of these nine is disability. However, achieving equality of opportunity for disabled people, to the extent that it is achievable, often requires different treatment [3]. Policymakers face the challenge of addressing economic inequality, which has been shown to lead to social exclusion and/or poverty. For instance, the poverty rate among people in families where someone has a disability is 8 percentage points higher than of those in families where no one is disabled [6]. To address this problem, governments rely on several indicators: household income, deprivation, employment, education, and social participation. Income, often the central measure used by the government, is used as a proxy for well-being, because it can be used to generate consumption and is positively correlated with standard of living [7]. Generally, a higher income allows more consumption which translates into a higher standard of living. However, Sen [8] argues that personal heterogeneities, such as a disability, affect not only the ability to generate an income (earning handicap) but also the ability to convert money into good living (conversion handicap). As clarified by Kuklys [9], “*standard monetary measures of individual welfare reflect only the first disadvantage* [and do not account] *for the second disadvantage (the higher consumption costs faced by these individuals)*.” As such, one limitation of current income-based analysis of poverty and inequality is to focus on the earning handicap resulting in the substantial underestimation of poverty and inequality for families with disabled members.

As in many other countries, the UK government recognises that disabled households need to cover extra costs, and thus, their income has to stretch further than the income of non-disabled households [4]. In other words, disabled people have additional costs to meet to achieve the same standard of living as a non-disabled counterpart. For example, someone with a learning disability might need special tutoring for school or a person with mobility impairments might require special transport arrangements and adaptations of the home. As a result, the UK government implemented several welfare benefits that are designed to help disabled people live more independently or finance support. Available welfare benefits include the Personal Independence Payment (PIP), the “new style” Employment and Support Allowance (ESA), the Attendance Allowance (AA), the Disability Living Allowance (DLA), and Universal Credit (UC). However, some of the benefits are mutually exclusive. For example, someone who receives the PIP or DLA cannot, at the same time, claim the AA.

This study investigates the extra costs of living faced by disabled households in the UK over the period 2013–2016. The findings suggest that the additional expenditure for disabled households to attain the same standard of living as their non-disabled peers has been increasing in the years between 2013 and 2016. The weekly extra costs, after adjustments for inflation, were £293 in 2013 at median income, which grows to £326 in 2016. These results are consistent with the previous studies, however, extend the current literature by displaying time trends.

We first begin by reviewing the existing literature and different approaches employed to estimate the extra costs faced by disabled people (see the section “Previous research”). The section “The standard of living method” introduces the standard of living approach. The section “Data and variables” will discuss the data set and variables used in the model. In the section “Estimation”, the estimation is presented. The section “Results” gives an overview of the results and robustness checks. The section “Discussion” presents our discussion and interpretation of the findings. Finally, the section “Conclusion” concludes.

## Previous research

There is a long history of research on the additional living costs linked to disability. Earlier studies employed various methods to estimate and adjust for the costs of disability (see Morciano et al*.* [10]; Antón et al*.* [11]; Berthoud et al. [5] for a review). Much of this work uses small-scale in-depth qualitative enquiry to evaluate the impact that various disabilities can have on individual lives. This “direct” approach estimates the disability costs mainly using a “survey” or “budget standard”. The survey approach attempts at asking disabled individuals (or experts) to identify disability‐related costs. See for example Martin and White [12], Thompson et al. [13], and Baldwin [14] for the UK. While intuitive and broadly used, the “direct” approach has caused some discussion regarding the accuracy of evaluation inferred from counterfactual and hypothetical situations in which respondents can accurately conceive the costs they would incur if they were living without (or with) disability. The budget standard approach attempts to identify a list (budgets) of minimum essential items (and associated costs) a household requires to achieve a minimum acceptable living standard. This approach has been extended (see, for example, Smith et al*.* [15] and Hirsch and Hill [16] for application to the UK) to the estimation of the extra costs of disability by identifying the particular budget for a disabled household to be compared with the budget of a non-disabled counterpart. While this approach appears more objective that the direct survey approach, estimates rely on the views of the experts and are prone to similar criticisms [17].

Other research has used a quantitative approach. It attempts at estimating “indirectly” the extra costs by fitting statistical welfare model to survey data and inferring the extra costs of disability from estimates of its parameters. The expenditure diary approach uses revealed preference methods to infer on the costs of disability from consumption patterns observed in survey data (see, for example, Jones and O’Donnell [18]; Matthews and Truscott [19]; and Baldwin [14] for the UK). While this is appealing in several contexts, the approach relies on the availability of good data and strong identification problems (see, e.g., Berthoud et al. [5]; Muellbauer [20]; Pollak and Wales [21]; Coulter et al. [22]; Banks et al. [23]; Deaton and Paxson [24]). An alternative is the life satisfaction approach, based on individuals’ reported satisfaction with their well-being using arbitrary numerical scales or judgments on the level of income believed necessary to reach a specified standard of living (see, for example, Adabbo et al. [25]). This approach has been subjected to conceptual and practical criticisms. From a conceptual viewpoint, a broad welfare measure raises issues on the “existence” and the “meaning” of such estimates from policy purposes (Hancock et al*.* [26]). From a practical viewpoint, there are issues due to the nature of assessments based on subjective perceptions and estimation issues coming from measurement errors (Morciano et al*.* [10]).

A standard of living approach is frequently adopted to overcome the limitations associated with the above-mentioned approaches. As described in the section “The standard of living method”, econometric models are used to fit an empirical standard of living to income curves for disabled and non-disabled counterparts and estimate the additional income that would be required to bring the disabled to the same standard of living as an otherwise identical non-disabled counterpart. Using a welfare concept limited to the idea of material living standards, it matches the rationale of realistic government policy to partially meet the material costs associated with disability better than approaches that use broader notions of happiness or life satisfaction. Berthoud et al*.* [5] pioneered the standard of living method that has been used in several countries (Mitra et al. [27] provide an excellent overview) and refined in multiple ways. Although there were some attempts to fit nonparametric (matching) models (see, for example, Hancock et al. [26]; Melnychuk et al. [28]; Solmi et al. [29]) most of the literature has used parametric approaches. Parametric approaches have been used in a single or structural fashion, with or without a latent factor or principal component specification (Morciano et al. [10]) to address the coursing nature of the standard of living and disability indicators collected in surveys. The majority of studies estimated extra costs using single cross-sectional data, with few attempts at measuring them longitudinally or to estimates trends using pooled cross-sections (see, for example, Cullinan et al. [30] for Ireland).

Table 1 summarises the previous estimates of extra costs of disability in the UK. We divide the estimates into four sub-groups: direct survey estimates, expenditure diary estimates, budget standard estimates, and standard of living estimates. The indirect survey method mostly used around the 1980s evinces considerable differences. Estimates range from £66 to £712. The Budget Standard estimates are among the highest (£1236 to £1434) of all surveyed papers. Relevant Standard of Living estimates for the United Kingdom range from £366 to £3576 depending on the definition and severity of disability.

**Table 1:** Overview of previous estimates of extra costs of disability

Study name	Data year	Geographical coverage	Approach	Extra £ per month in 2020 prices
1. Direct survey approach estimates				
Martin and White [12]	1985	Great Britain (representative)	Face-to-face interview	£74
Thompson et al*.* [13]	1988	Various parts of Great Britain (non-representative)	Telephone survey	£712
2. Expenditure diary approach estimates				
Matthews and Truscott [19]	1985	Great Britain (non-representative)	Follow-up interview to OPCS survey	£66
Baldwin [14]	1978	Great Britain (non-representative)	Interviews with disabled families	£155
3. Budget standard approach estimates				
Smith et al*.* [15]	2004	Derby, Birmingham, and Nottingham	Case studies of disabled people	Low–medium needs: £1236 Intermittent needs: £1286 High-medium needs: £1434
4. Standard of living approach estimates				
Berthoud et al*.* [5]	1985	Great Britain (representative)	Regression analysis of data from the OPCS survey and Family Expenditure Survey	£366
Zaidi and Burchardt [31]	1996/97	United Kingdom (representative)	Regression analysis of data from the FRS 1996/97 data and the Disability Follow-Up survey	Low severity: £118–£629 Medium severity: £360–£1893 High severity: £681–£3576
Touchet and Morciano [46]	2015/16 [2016/17]	United Kingdom (representative)	Regression analysis of data from the FRS 2015/16 [2016/17] data	£636 [£639]

## The standard of living method

The central premise of the standard of living method is that disabled people experience lower living standards than their healthy equivalent with the same level of income. This stems from the assumption that households with a disabled individual have to divert some of their income to satisfy disability-related needs. The diversion of resources takes away from products and services that would otherwise be purchased to improve their general standard of living.

**Fig. 1:**
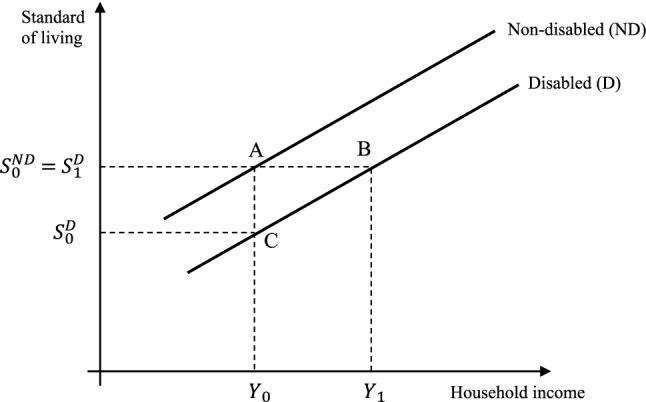
Graphical representation of the standard of living approach

Figure 1 plots the curves relating standard of living (*S*) and income (*Y*) for a disabled (*D*) households and for a comparable non-disabled (ND) household. Exemplifying the relationship between income and standard of living, the curve for non-disabled households is above the curve for disabled households. The distance ‘AB’ measures the additional income that the disabled household would require to achieve the same standard of living of the non-disabled counterpart.

Following Zaidi and Burchardt [31], the model can be expressed as:

1 $$S=\alpha Y+\beta D+\gamma X+k,$$

where $S$ is the standard of living indicator, $Y$ is the household income, $D$ is the disability status of household (1 = yes, 0 = no), $X$ is the vector of household level characteristics, $a, \beta , \gamma$ is the equation parameters, $k$ is the intercept term (a constant absolute minimum standard of living; the standard of living for households without a disabled member when income is zero).

From Eq. (1), we can estimate the additional expenditure of disability, $E$. If we assume that with extra income, *Y* + *E*, disabled households can attain the same standard of living as non-disabled households, then this condition applies: ${S}_{\mathrm{disabled}}={S}_{\mathrm{non}-\mathrm{disabled}}$. We can then solve for *E*

2 $$\begin{gathered} \underbrace {{\alpha \left( {E + Y} \right) + \beta \left( 1 \right) + \gamma X + k}}_{{S_{{{\text{disabled}}}} }} = \underbrace {\alpha Y + \beta \left( 0 \right) + \gamma X + k}_{{S_{{\text{non - disabled}}} }} \hfill \\ E + Y + \frac{\beta }{\alpha } = Y \hfill \\ E = - \frac{\beta }{\alpha }. \hfill \\ \end{gathered}$$

Equation (2) can be verified by calculating the total differential, $E=\frac{dY}{dD}=-\frac{\beta }{\alpha }$. In other words, the extra cost of disability expressed as a percentage of income can be obtained by dividing the disability coefficient by the estimated log income coefficient. This is an established methodology which has been used in several other recent studies (see, for example, Zaidi and Burchardt [31]; Cullinan et al*.* [30]; Ipek [32]).

This approach simplifies the model, because it assumes the relationships between standard of living and income (*S*, *Y*) for households with and without disabled member are linear and parallel to each other which implies that the extra costs of disability would be the same for any level of income. However, the relationship between standard of living and income could take a non-linear form, which can be analysed empirically. Morciano et al. [10] and Zaidi and Burchardt [31] present an extended discussion of the importance of using appropriate functional forms to model the (*S*, *Y*) relation. To allow comparison with the previous studies, we assume a standard linear-natural log relationship of *S* with *Y*. The implication of this specification is that the marginal returns of income in terms of the standard of living decrease as income rises, that is to say, an extra £1 in income increases the standard of living of a poor person relatively more than for a wealthy person.

## Data and variables

This paper uses repeated cross-sectional data from the Family Resources Survey (FRS) from 2013 to 2016. We were limited by the change of the disability definition and inclusion of three deprivation questions that contribute to our standard of living index.^1^ The chosen time frame allows meaningful and direct comparisons between the years, because the relevant variables do not change in these years. The FRS is an annual representative survey which has been running in Great Britain since 1992. The survey collects data on the income and living circumstances of households and families in the UK. The average response rate of 60% is consistent and high for this kind of survey. It is calculated as the ratio of the number of fully co-operating households to the number of eligible households. The sample size is approximately 20,000 households per year. This paper follows the structure for the standard of living questions in the FRS and hence computes all variables at a family/household level. The set of variables used in the model are briefly explained below.

### Standard of living indicator

The key dependent variable in all models is an indicator of the latent (unobservable) standard of living of each household. Zaidi and Burchardt [31] provide a more detailed discussion of the standard of living variable. Following previous studies (see, for example, Berthoud et al. [5]; Cullinan et al. [30]), this paper constructs a composite indicator of standard of living. The indicator is derived as a function of ten questions regarding ownership or deprivation (set of questions in Appendix 1). The questions take the value 0 if the respondent cannot afford the activity/good and takes the value 1 otherwise. The composite indicator of standard of living is derived by scaling up the total score. The index is designed to ensure a reasonably equal distribution within each step. The standard of living indicator follows the pattern that if a household scores either 0, 1, or 2, the composite index will assume a value of 1. Scores of three and four correspond to a value of 2. If a household scores 5, the composite index will exhibit a 3*.* A composite index value of 4 equals six and a value 5 matches seven as a score*.* The highest value of 6 in the composite index equals eight positive responses*.* For instance, a household that answers each question with *‘yes’* scores 6 and has a high standard of living, because the family has the resources to afford all items. However, a family with less income may not be able to afford the same number of items and thus scores lower on the standard of living scale. This demonstrates a sensitivity of the indicator to changes in available resources. The design of the indicator implicitly assumes that each activity/good is of equal importance. This is done for reasons of simplicity, because determining the importance of each activity/good for each individual household would not be possible. The distribution for the standard of living variable is relatively constant over the analysed time period (see Appendix 3).

### Disability variable

The definition and severity of disability plays a significant role in the level and nature of public welfare programmes. Therefore, it is necessary to provide a definition of disability that is appropriate for use in the empirical analysis. The UK government uses the Equality Act 2010 as their starting point for a disability definition. A disability is defined as follows:

A person (P) has a disability if—

- P has a physical or mental impairment, and
- the impairment has a substantial and long-term adverse effect on P’s ability to carry out normal day-to-day activities [33].

Substantial ensures that only non-trivial or major impairments are covered. Impairments include, but are not limited to, long-term conditions such as diabetes, deafness, or paralysis. Mental impairments cover learning difficulties such as attention-deficit hyperactivity disorder (ADHD) or dyslexia but also other mental health conditions such as bipolar disorder. Some disabilities are automatically included and do not need to explicitly demonstrate the substantial adverse effects on daily lives (i.e., HIV or multiple sclerosis). The Equality Act definition is the basis for identifying disabled households and estimating their extra costs herein.

The relevant measure of disability considered in this paper is the yes/no response to the question “*Whether [respondent] has a disability (the Equality Act 2010—wider def)?*” in the FRS. The binary nature of this variable has the advantage that it is easy to interpret and consistent across all years. Other papers have experimented with accounting for the level of severity of the disability. However, the questions in the FRS change over survey years which means that it is difficult to maintain consistency across all years. Therefore, we focus on the dummy variable for the presence of disability. Given the recent change of the definition and the necessary changes in the survey, the new definition if first adapted in the 2012/2013 questionnaire.

### Other variables

The income variable is based on total household income which is an accumulation of a number of different income indicators. The total household income reflects the available resources to a given household, including disability and social security benefits, in addition to income from investments, bursaries, or pensions. Following other UK studies, we subtract housing cost from the total household income (see Zaidi and Burchardt [31]). It should be noted that direct taxes have not been removed. In Appendix 3, the distribution of the income variable shows that incomes are relatively consistent over time with a small dip in 2015/2016.

The model also includes several other explanatory variables. The inclusion of further variables was directed by other papers and their performance in the model. Appendix 2 demonstrates that all explanatory variables are statistically significant. Tenure is known to cause differences in standard of living when incomes are equal. For example, someone who owns a house and makes £1000 per month will have a higher standard of living as someone who makes the same amount but rents an apartment. This is equally true for regional differences. For instance, someone with £1000 per month in London, one of the most expensive cities in the world, will have a lower standard of living than someone living in Manchester. The number of dependent children in a household, as well as the general household composition, play a significant role in determining the standard of living. The more people are in a household, the more the income has to stretch. The age of the household reference person is also important, because income is expected to rise with age until it declines after retirement. Table 2 summarises the variables of the model.

## Estimation

This paper uses a multivariate modelling approach to analyse the underlying relationship between standard of living and income, disability, and other characteristics. Given the list of variables in Table 2, the model estimated is

$${S}_{i}^{*}={\beta }_{0}+{{\beta }_{1}\mathrm{inc}}_{i}+{{\beta }_{2}D}_{i}+{{\beta }_{3}\mathrm{tenure}}_{i}+{{\beta }_{4}\mathrm{region}}_{i}+{{\beta }_{5}\mathrm{child}}_{i}+{{\beta }_{6}\mathrm{age}}_{i}+{{\beta }_{7}\mathrm{hhcomp}}_{i}+{\varepsilon }_{i}.$$

**Table 2:** Variable definitions and summary statistics for selected variables

Variable	Definition	Summary statistics
Dependent variable		Mean for each year (SD)
Standard of living ($${S}_{i})$$	Standard of living indicator taking integer values from 1 to 6	[13/14] 4.26 (1.88) [14/15] 4.34 (1.85) [15/16] 4.41 (1.82) [16/17] 4.54 (1.76)
Disability status		% of households in sample
Disability (${\mathrm{dis}}_{i})$	= 1 if household is disabled, = 0 otherwise	[13/14] 26.91 [14/15] 28.62 [15/16] 30.31 [16/17] 30.35
Income variable		Median for each year
AHC income, log (${\mathrm{inc}}_{i})$	Natural log of after housing cost income	[13/14] £472 [14/15] £494 [15/16] £484 [16/17] £509
Other explanatory variables		Mean for each year
Tenure (${\mathrm{ten}}_{i})$	6 tenure dummy variables	N/A
Region (${\mathrm{reg}}_{i})$	12 regional dummy variables	N/A
Children (${\mathrm{child}}_{i})$	Number of dependent children in the household	[13/14] 0.77 [14/15] 0.75 [15/16] 0.75 [16/17] 0.75
Age (${\mathrm{age}}_{i})$	Age band of household reference person	[13/14] 42.9 [14/15] 43.0 [15/16] 42.7 [16/17] 43.0
Household composition (${\mathrm{hhcomp}}_{i})$	17 household dummy variables	N/A

Given the standard of living variable is an ordinal outcome, and following previous studies, we apply an ordered logit model (see Cullinan et al*.* [30]; Indecon [34]). The model is based on an underlying latent variable ${S}^{*}$, such that

$$\begin{gathered} S = 1\quad {\text{if}}\quad S^{*} \le j_{1} \hfill \\ S = 2 \quad {\text{if}}\quad j_{1} \le S^{*} \le j_{2} \hfill \\ \cdots \hfill \\ S = N\quad {\text{if}}\quad S_{n - 1}^{*} \ge j_{n - 1} , \hfill \\ \end{gathered}$$

with ${j}_{0}, {j}_{1}, \dots , {j}_{n-1}$ being the thresholds that need to be crossed over to reach the next higher level of standard of living; they need not be equidistant [35]. *S* represents a composite index of the number of positive answers of households. To allow for some variation in preferences each question adds to the count of S in the same way. A more detailed discussion of the ordered logit model can be found in Zaidi and Burchardt [31] in a standard of living context or Greene and Hensher [36] for a more general discussion.

## Results

Table 3 illustrates the results of the estimation separately for each of the 4 years between 2013 and 2016. The analysis is conducted separately for each year, because the FRS produces cross-sectional data. The upper half of the table shows the marginal effects^2^ on selected included variables. In particular, the disability and income variable are highlighted. The income variable is modelled using the *natural log* of income. The lower half of the table provides the extra costs estimates as a percentage of income and in £ per week in prices of the respective year and 2020 prices. We also report the associated 95% confidence intervals in the table. The table also includes McFadden’s Pseudo *R*-squared to give an indication of the explanatory power of the model. The goodness of fit for the model is within the expected range and close to similar regressions (see, for example, Indecon [34]). In each year, the disability and income coefficient, including the corresponding standard error, are presented. The estimation of extra costs is illustrated by the ratio of disability and income coefficients.

**Table 3:** Summary of results

	2013–2014	2014–2015	2015–2016	2016–2017
AHC income, log	1.085*** (0.019)	0.996*** (0.019)	0.997*** (0.020)	0.991*** (0.020)
Disability	− 0.519*** (0.026)	− 0.500*** (0.026)	− 0.517*** (0.027)	− 0.520*** (0.027)
Tenure	Y	Y	Y	Y
Region	Y	Y	Y	Y
Children	Y	Y	Y	Y
Age	Y	Y	Y	Y
Household composition	Y	Y	Y	Y
Pseudo *R*^2^	0.14	0.13	0.14	0.14
Extra costs estimate as % of income ($\beta$)	47.8	50.2	51.9	52.5
CI lower bound	− 0.427	− 0.446	− 0.461	− 0.467
CI upper bound	− 0.530	− 0.558	− 0.576	− 0.583
Median income, £pw	£531	£552	£543	£566
Extra costs, £pw	£254	£277	£282	£297
[95% C.I.]	[£227 to £281]	[£246 to £308]	[£250 to £313]	[£264 to £330]
Extra costs, £pw in 2020 prices	£293	£312	£315	£326
[95% C.I.]	[£262 to £325]	[£277 to £347]	[£280 to £350]	[£290 to £362]
Observations	29,881	29,313	27,929	28,459

Sources: FRS 2013/2014 [1], 2014/2015 [37], 2015/2016 [38], 2016/2017 [39]

Ordered logistic regression, dependent variable: standard of living indicator. Standard errors are in parentheses. The FRS tends to include extreme values which affect the mean, and therefore, median income is used (FRS [1]). 95% confidence intervals calculated using the ‘*nlcom*’ command in *Stata*. More detailed data in Appendices 1, 2, and 3

Statistical significance at *** 1% level ** 5% level * 10% level

The estimation results in Table 3 demonstrate that disability is negatively correlated with the standard of living and statistically significant at the 1% level over the whole observation period. The coefficient mean is – 0.51 with only slight deviations in the individual years. The regression also clearly establishes a positive relationship between income and standard of living, which is $1.02$ on average over the 4 years. The extra cost of disability, calculated by Eq. (2), stated as a percentage of income climbs from 47.8% in 2013/2014 to 52.5% in 2016/2017. The average extra cost over 4 years is 50.6% of weekly median income. The confidence intervals overlap between the years; however, looking at the lower and upper bound, we can see that they also exhibit an upward trend. While the confidence intervals overlap, there is a general upwards trend. At best, this indicates that there has been no positive change to reduce the extra costs required. This paper adopted median income instead of mean income as recommended by the FRS [1], because the measure is less susceptible to extreme values. Additionally, the income is presented gross of direct taxes which increases the estimates. The implied extra costs of disability range from £254 in 2013 to £297 in 2016 per week. Despite the variation between the years, the estimate is regularly found to be statistically significant. Thus, disabled households face a sizeable financial burden. As the calculation are at median income, there will be some households who face even higher costs due to their disability. In sum, the estimates in Table 3 are in all years consistent with the hypothesis that disabled people have to pay more to attain the same standard of living as non-disabled families with the same income.

### Robustness checks

To check the robustness of our estimates, we run several alternative specifications. First, we use household weights to produce weighted estimates. Second, we check estimate an ordinary least squared model (OLS).

#### Weights

We use household weights which ensures that the FRS can be used to produce estimates which are representative of the UK. Our results show that the inclusion of weight does not change our results significantly. Table 4 shows a brief comparison of the extra cost estimates obtained using the weighted sample and unweighted sample. However, as expected with the inclusion of weights, the standard errors increase significantly, but the same statistical significance is achieved in all cases (*p* < 0.001).

#### OLS specification

Our OLS specification produces qualitatively similar results. The small differences may arise, because the OLS model implicitly adopts no upper limit to the standard of living. The ordered logit regression, on the other hand, implicitly assumes that a value of six for the composite indicator is the highest attainable standard of limit. In line with our previous estimates, the OLS specification shows an upward trend from 2013/2014 to 2016/2017. However, we can observe a small drop in this trend in the 2015–2016 data (Table 5).

**Table 4:** Logistic regression—weighted sample

	Without weights (%)	Std. error	With weights (%)	Std. error
2013–2014	47.8	0.0263	45.4	0.0309
2014–2015	50.2	0.0285	51.2	0.0352
2015–2016	51.8	0.0293	49.2	0.0357
2016–2017	52.5	0.0296	52.0	0.0383

**Table 5:** OLS specification

Dependent variable	Standard of living			
	2013–2014	2014–2015	2015–2016	2016–2017
AHC income, log	0.796*** (0.013)	0.713*** (0.013)	0.699*** (0.013)	0.675*** (0.013)
Disability	− 0.420*** (0.021)	− 0.410*** (0.021)	− 0.401*** (0.020)	− 0.391*** (0.019)
Observations	29,881	29,313	27,929	28,459
Adjusted *R*^2^	0.358	0.331	0.340	0.337
Extra costs estimate as % of income	52.8	57.5	57.3	57.9

Statistical significance at *** 1% level ** 5% level * 10% level

#### Pooled regression and interactions

We also estimated interaction models based on pooled data. In separate models, we interacted: (i) the disability status indicator with year dummies; (ii) income with year dummies; and (iii) disability with income and with year dummies. In all specifications, we additionally included year dummies. We obtained the additional cost of living required using Stata’s ‘*nlcom*’ command. The results consistently showed a general upward trend in the extra costs required, but there was very little statistical significance, similar to the main analysis (see Appendix 2).

## Discussion

As shown in Table 3, the extra costs in real and nominal terms increased between 2013 and 2016, albeit not statistically significantly so. There are many reasons to believe that extra costs are slowly increasing over time. Since 2010, there have been significant changes to the welfare system in the UK. The measures included, inter alia, social security budget cuts, and the removal of benefits for a wide range of people. A likely driver for increased additional expenditure was the introduction of the Welfare Reform and Work Act 2012, which was an austerity measure to reduce the amount of welfare spending in the UK. However, we cannot empirically test for that here, and hence, this is a speculative explanation. Kennedy [40] suggests that disabled people have been negatively and disproportionately affected by the changes to the welfare system. The Act introduced a benefit cap limiting the total welfare paid out to £384.62 a week for a couple and £257.69 a week for a single person. Ken Butler [41], a welfare rights and policy adviser, summarises the austerity measures: “PIP was […] designed to reduce disability benefit spending by 20%. ESA [Employment and Support Allowance] has been reduced by around £30 per week for many new claimants since April 2107. Universal Credit also excludes the severe disability premium worth around £65 per week to those formerly entitled to it.” This has been underlined by the Equality and Human Rights Commission [42] which estimated that the switch from the Disability Living Allowance to PIP would decrease the number of recipients by 28%. Another study by the Institute for Fiscal Studies projected that the benefit cap equals an 8% reduction in real monetary terms in the period from 2012 to 2019 [43]. These factors negatively impact the incomes of disabled people and, thus, increase the extra costs of disability.

Additionally, we use the Standard of Living approach which only incorporates data on what is spent and not necessarily what is needed. It is also sensitive to the choice of disability, income, and standard of living variables used. A discussion of potential limitations to the Standard of Living approach is provided in the systematic review of Mitra et al. [27]. However, in our analysis, we use a consistent set of variables, and hence, these issues are potentially mitigated against in that they are at least internally consistent.

Another problem that came with the changes to the welfare system was the introduction of a 6-month qualifying period for PIP. Especially newly disabled people are disadvantaged by this prerequisite, because they have to wait a significant amount of time before they can access support payments. For example, a new wheelchair user faces many start-up costs to adapt to the new situation. If the person lives in a house, they will have to install a stair lift, and the use of certain goods such as transport cost or insurance payments will inevitably go up. While some security benefits are designed to address the early period, the Work and Pensions Committee [44] does not believe that they are sufficient to cover the rising costs. The changes to the UK welfare system clearly have had adverse effects on disabled households and increased their expenditures further.

### Comparison with previous estimates

The comparison with previous results is often difficult because of different focuses, methodologies, and data sets. A direct comparison is, therefore, only meaningful if those parameters are reasonably similar. The literature review shows that two authors have used a similar approach with the same data set in the UK. Therefore, this comparison will be restricted to the papers by Zaidi and Burchardt, as well as the charity Scope.

The results derived by Zaidi and Burchardt while similar in the approach are more granular and differ in their analysis by household composition (single and couple) and age group (pensioner and non-pensioner). It should be noted that the pooling of all household in this paper can skew the data, because certain household may produce extreme values which increase the estimate. However, in 2020 prices, Zaidi and Burchardt’s range of estimates is comparable to the estimates presented above. The average cost estimate of this paper is £312 [2020 prices] per week and thus falls into the medium to high severity estimates provided by Zaidi and Burchardt. This was to be expected, because this paper uses a dummy variable for the disability status. The existence of a disability follows the definition of the Equality Act 2010 and can therefore sometimes disregard less severe disabilities. The OPCS severity scores of disability utilised by Zaidi and Burchardt, for example, would include an older man that is short sighted and has difficulty to read ordinary newspaper print and follow a conversation against background noise (see Martin et al. [45]), whereas the Equality Act 2010 would not recognise him as disabled. Moreover, the reasons presented in the paragraph before suggest that it was likely to see an increased expenditure year over year. In sum, the results presented here are in line with the previous estimates by Zaidi and Burchardt.

The recent publication by the Scope charity (Touchet and Morciano [46, 47]), which employed a structural equation model found that on average disabled people in the UK face extra costs of £581 a month related to their disability. However, in the highest quintile, the mean extra cost reaches £1675 for disabled adults. The analysis utilises the same FRS data set that this paper uses; although, there are some differences in the modelling that can explain the variation in estimates. The Scope paper (ibid) computes four elements to estimate extra costs: “standard of living index, adult disability index, children disability index [and] income associated with levels of standard of living, while controlling for socioeconomic factors.” Given the addition of child disability indicators and interaction terms to the model, there are some considerable modifications in the computation. These differences could explain the differences in outcome. Generally, both estimates show a significant financial burden to disabled households.

While there is an increasing trend in the additional money required to ensure an equal standard of living, the differences over time are not statistically different from each other. However, this implies that the gap is not narrowing, as was hoped by UK Government Policy.

### Policy recommendations

The problem of tackling extra costs requires action from society and the government alike. However, the policy recommendations presented in this paper will focus on the government part. In the following paragraphs, this paper outlines two possible ways for the government to ease the financial burden on disabled households in the UK. A detailed discussion of more comprehensive policy suggestion would go beyond the scope of this paper; however, interested readers should consult one of the numerous foundations and charities in the UK that specialise in disability policymaking (see, for example, Scope charity, Disability Rights UK, Equalities National Council, or the Joseph Rowntree Foundation).

At the heart of government action is the welfare system. To address the extra costs better, the government must be able to assess the eligibility of applicants more reliably and adapt the support payments to the needs. Thus, the main area of reform must be the PIP. Even after the receipt of PIP and other benefits, this paper showed that disabled people still face high extra costs. That is because the PIP assessment fails to determine the needs accurately. A poll carried out by the Scope charity found that more people 36% of people disagreed that the assessment process accurately captured the additional costs they faced. Therefore, the government must reform the PIP assessment to ensure that the extra costs are correctly recorded.

Another approach to tackle disability-specific costs would be working closely with disability organisations, the Competition and Market Authority, and disabled individuals to understand and subsequently close market vulnerabilities. With respect to the example from the section “Previous research”, people with diabetes are especially affected by the ‘sugar tax’ which was meant to crack down on high sugar levels in soft drinks. However, people with diabetes sometimes precisely need this high level of sugar to tackle low blood glucose levels. Therefore, the government should work together with several bodies to identify the areas in which disabled persons face vulnerabilities. While this is a specific example, there are many more examples from different disabilities out there. In sum, the government should introduce assistance to address those vulnerabilities and take this extra financial burden from the shoulders of disabled people.

### Research agenda

To design successful future policies, further research is necessary. In the future, more detailed data collection methods can provide a better starting position for analyses. While the FRS provides a good data basis, there are many questions that have a high proportion of missing values which limits the analytical options. The FRS data set is also unspecific, so to gain comprehensive quantitative evidence on disability-related questions, it would be a good idea to re-introduce the disability follow-up questionnaire that Zaidi and Burchardt were able to use for their analysis. Another improvement for estimates would be if there were longitudinal or time series data sets, because they allow costs to be tracked over time. Furthermore, the research should also be participatory. An integrative approach that lets experts design the follow-up questionnaire could ensure a higher quality of data. Moreover, researchers can identify potential issues early in the data collection process. Generally, the quality of the data analysis rises with the quality of the underlying data.

Additionally, future research should consider the Goods and Services and Goods and Services Required approaches (see Mitra et al*.* [27] for further details). This would enable a detailed comparison to the standard of living approach more widely used in the literature.

## Conclusion

This paper reinforces the hypothesis that disabled households in the UK face additional expenditure to attain the same standard of living as households without a disabled member. To estimate the extra costs the so-called standard of living approach is utilised. The modelling method is based on papers by Berthoud et al. or Zaidi and Burchardt. The findings suggest that after disability benefits households with a disabled member faced extra cost of 47.8% of median income per week in 2013, which equals £293 in 2020 monetary terms. The next year the extra cost estimate as percentage of income increased to 50.2% which represents £312 in 2020. In 2015, there was another increase to 51.2% or £315. The last year of observations estimated the extra costs at 52.5% which equals £326. Overall, the trend line for the results shows a positive trend, albeit not statistically significant, which indicates that the extra costs increased over the period of observation. At best, the extra costs required have not narrowed, which is indicative of policy that is not working for these disabled households. In reality, this means that it gets harder and harder for disabled households to achieve the same standard of living as their non-disabled counterparts.

In comparison with the previous studies, the estimates are comparable albeit to the higher severity categories. While other papers used a severity score, this paper employed a disability dummy variable which was based on the disability question in the FRS. Moreover, this paper did not differentiate the estimate for different household compositions which could lead to a higher estimate. However, the results can still supply an important basis for future policy decisions. The paper made two main policy recommendations: (1) reform the assessment test for the Personal Independence Payment, and (2) close market vulnerabilities for disabled households by working closely with disabled persons and other relevant organisations. To ensure the success of these policies, the paper suggests conducting more research in the future to guide the policy process.

## Figures and Tables

**Fig. 2:**
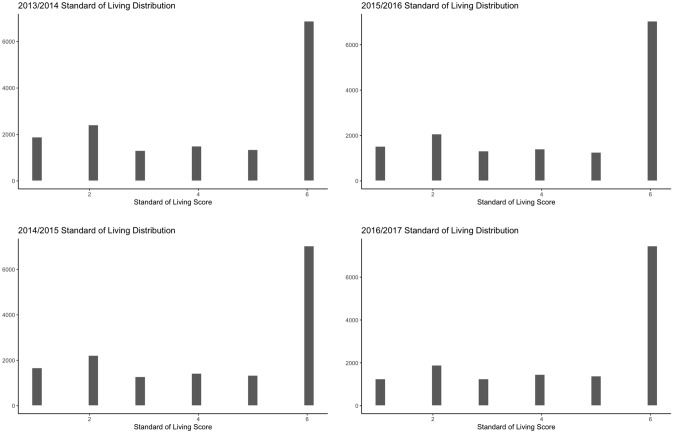
The distribution of the standard of living variable over time

**Fig. 3:**
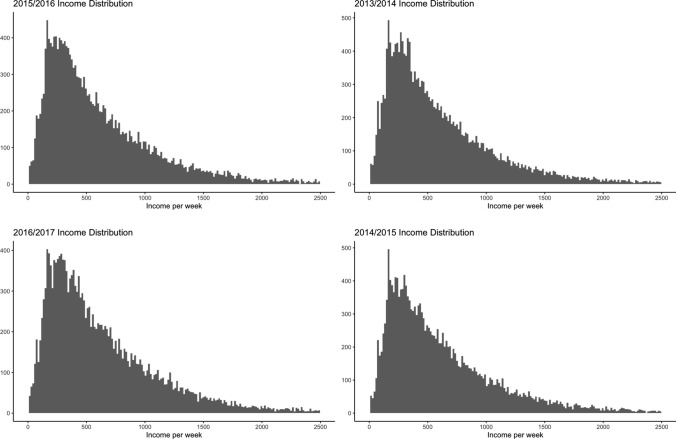
The distribution of income over time

## Data Availability

The data are available from the UK Data Service.

## Appendix 1

This section looks at a large number of items that can be associated with standard of living. For the purpose of this study, hardship is defined by the respondents’: ability to afford a number of items that most other people agree families ought to have; their other ‘unmet needs’; and whether they are managing their money and staying clear of problem debts—that is debts they cannot repay and are ‘getting behind with the repayments These questions will be used to gain a better understanding of people’s living standards and the spending choices that they make. No single one of these items is a very adequate measure, but taken together they add up to a very sensitive measure of family material well—being or hardship. The series of questions which determine hardship are either factual or opinion based. For them to effectively determine hardship and deprivation the answers must reflect the respondent’s interpretation of the question. Do not attempt to guide or re-phrase the question. If the respondent does not understand what is being asked (for instance they are unclear about what we mean by ‘all weather shoes’), simply repeat the question and ask them to answer it to the best of their ability. Please do not give your translation of a phrase or question. The questions will only be asked of ONE adult in the benefit unit. The respondents can then answer together if they wish. The computer will randomly select the adult required to answer the deprivation questions (Family Resources Survey [FRS], 2013–2014).

1. Enough money to keep your home in a decent state of décor?
2. Do you regularly participate in a hobby or leisure activity?
3. Hold away from home one week a year + not staying with rels?
4. Household contents insurance?
5. Do you get friends or family around for a drink or meal at least once a month?
6. Make savings of £10 a month or more?
7. Do you have two pairs of properly fitting shoes, including a pair of all weather shoes?
8. Replace any worn out furniture?
9. Replace or repair broken electrical goods?
10. Money to spend each week on yourself, not on your family?

## Appendix 2

See Table 6.

**Table 6:** Full regression results for the ordered logit model

Dependent variable	Standard of living				
	2013/2014	2014/2015	2015/2016	2016/2017	Pooled regression
AHC income, log	1.085*** (0.0193)	0.996*** (0.0190)	0.997*** (0.0199)	0.991*** (0.0199)	1.016*** (0.00972)
Disability	− 0.519*** (0.0263)	− 0.500*** (0.0262)	− 0.517*** (0.0267)	− 0.520*** (0.0267)	− 0.512*** (0.0132)
Children	− 0.440*** (0.0611)	− 0.268*** (0.0604)	− 0.561*** (0.0673)	− 0.375*** (0.0635)	− 0.399*** (0.0314)
North West	− 0.0339 (0.0715)	− 0.102 (0.0679)	0.0848 (0.0676)	0.0251 (0.0669)	− 0.0105 (0.0340)
Yorks and the Humber	0.0701 (0.0747)	− 0.0327 (0.0726)	0.126* (0.0711)	0.125* (0.0709)	0.0702* (0.0359)
East Midlands	0.0230 (0.0756)	0.170** (0.0740)	0.190** (0.0753)	0.102 (0.0731)	0.122*** (0.0370)
West Midlands	− 0.0317 (0.0733)	− 0.0667 (0.0698)	0.210*** (0.0715)	0.237*** (0.0683)	0.0809** (0.0351)
East of England	0.0386 (0.0743)	0.131* (0.0721)	0.296*** (0.0713)	0.276*** (0.0714)	0.178*** (0.0359)
London	0.0111 (0.0707)	− 0.0612 (0.0686)	0.131* (0.0690)	− 0.155** (0.0668)	− 0.0185 (0.0342)
South East	0.0957 (0.0699)	0.00189 (0.0677)	0.330*** (0.0682)	0.202*** (0.0675)	0.154*** (0.0339)
South West	0.135* (0.0764)	0.00587 (0.0732)	0.283*** (0.0743)	0.188** (0.0741)	0.155*** (0.0370)
Wales	− 0.0348 (0.0819)	0.166** (0.0843)	0.219*** (0.0836)	0.0680 (0.0856)	0.107** (0.0415)
Scotland	0.0697 (0.0687)	0.194*** (0.0660)	0.345*** (0.0672)	0.146** (0.0647)	0.189*** (0.0331)
Northern Ireland	− 0.0575 (0.0710)	0.0825 (0.0691)	0.174** (0.0688)	0.136** (0.0679)	0.0784** (0.0343)
Age 20–24	0.302* (0.170)	− 0.200 (0.128)	− 0.373*** (0.132)	0.0765 (0.116)	− 0.0527 (0.0656)
Age 25–29	0.233 (0.168)	− 0.214* (0.125)	− 0.509*** (0.134)	− 0.0144 (0.115)	− 0.127* (0.0651)
Age 30–34	0.283* (0.167)	− 0.142 (0.125)	− 0.470*** (0.134)	0.00575 (0.115)	− 0.0767 (0.0649)
Age 35–39	0.177 (0.168)	− 0.238* (0.125)	− 0.463*** (0.135)	− 0.0659 (0.116)	− 0.142** (0.0653)
Age 40–44	0.101 (0.167)	− 0.275** (0.124)	− 0.499*** (0.133)	− 0.193* (0.114)	− 0.213*** (0.0646)
Age 45–49	0.0514 (0.166)	− 0.409*** (0.122)	− 0.683*** (0.131)	− 0.258** (0.112)	− 0.317*** (0.0638)
Age 50–54	− 0.192 (0.166)	− 0.594*** (0.122)	− 0.829*** (0.131)	− 0.222** (0.111)	− 0.466*** (0.0635)
Age 55–59	0.0502 (0.167)	− 0.452*** (0.122)	− 0.750*** (0.132)	− 0.168 (0.112)	− 0.331*** (0.0640)
Age 60–64	0.106 (0.169)	− 0.281** (0.126)	− 0.417*** (0.135)	0.130 (0.116)	− 0.114* (0.0657)
Age 65–69	− 0.495** (0.193)	− 1.358*** (0.148)	− 1.308*** (0.165)	− 0.934*** (0.148)	− 1.040*** (0.0793)
Age 70–74	− 0.743*** (0.204)	− 1.232*** (0.175)	− 1.335*** (0.192)	− 0.900*** (0.168)	− 1.062*** (0.0897)
Age 75 or over	− 0.219 (0.198)	− 0.948*** (0.161)	− 1.727*** (0.169)	− 0.778*** (0.159)	− 0.917*** (0.0835)
One female adult, no children, under pension age	− 0.0950 (0.0741)	− 0.00353 (0.0744)	0.149** (0.0736)	− 0.0340 (0.0751)	0.0132 (0.0370)
Two adults, no children, one over pension age	− 1.248*** (0.117)	− 0.813*** (0.119)	− 0.518*** (0.127)	− 1.032*** (0.125)	− 0.905*** (0.0605)
Two adults, no children, both under pension age	− 0.250*** (0.0573)	− 0.175*** (0.0577)	− 0.0418 (0.0557)	− 0.202*** (0.0603)	− 0.159*** (0.0288)
Three or more adults, no children	− 1.815*** (0.0607)	− 1.745*** (0.0606)	− 1.570*** (0.0592)	− 1.816*** (0.0632)	− 1.727*** (0.0303)
One adult, one child	− 0.501*** (0.105)	− 0.558*** (0.105)	− 0.226** (0.109)	− 0.531*** (0.111)	− 0.460*** (0.0536)
One adult, two children	− 0.517*** (0.154)	− 0.540*** (0.158)	0.149 (0.166)	− 0.404** (0.161)	− 0.351*** (0.0794)
One adult, three or more children	− 0.281 (0.241)	− 0.771*** (0.244)	0.456* (0.263)	− 0.367 (0.249)	− 0.274** (0.124)
Two adults, one child	− 0.277*** (0.0875)	− 0.344*** (0.0882)	0.190** (0.0925)	− 0.215** (0.0925)	− 0.171*** (0.0449)
Two adults, two children	0.117 (0.138)	− 0.0190 (0.137)	0.679*** (0.149)	0.0744 (0.144)	0.194*** (0.0706)
Two adults, three or more children	0.201 (0.217)	− 0.265 (0.217)	0.889*** (0.238)	0.0501 (0.228)	0.183 (0.112)
Three or more adults, one child	− 1.517*** (0.0917)	− 1.627*** (0.0921)	− 1.108*** (0.0983)	− 1.790*** (0.0957)	− 1.515*** (0.0469)
Three or more adults, two children	− 1.379*** (0.148)	− 1.402*** (0.149)	− 0.626*** (0.158)	− 1.559*** (0.154)	− 1.257*** (0.0758)
Three or more adults, three or more children	− 1.148*** (0.235)	− 0.944*** (0.247)	− 0.250 (0.255)	− 1.104*** (0.254)	− 0.925*** (0.123)
Buying with mortgage	− 0.353*** (0.0350)	− 0.283*** (0.0357)	− 0.212*** (0.0382)	− 0.243*** (0.0380)	− 0.278*** (0.0183)
Part own, part rent	− 0.910*** (0.156)	− 0.688*** (0.160)	− 0.914*** (0.138)	− 0.502*** (0.174)	− 0.776*** (0.0772)
Rents	− 1.539*** (0.0380)	− 1.460*** (0.0383)	− 1.469*** (0.0399)	− 1.511*** (0.0390)	− 1.492*** (0.0193)
Rent-free	− 0.794*** (0.124)	− 0.796*** (0.119)	− 0.680*** (0.125)	− 0.714*** (0.130)	− 0.756*** (0.0621)
Squatting		14.52 (729.2)			13.86 (523.1)
2014					0.0909*** (0.0162)
2015					0.221*** (0.0165)
2016					0.341*** (0.0165)
Observations	29,881	29,307	27,929	28,459	115,582

**p* < 0.1; ***p* < 0.05; ****p* < 0.01

## Appendix 3

See Figs. 2 and 3.

## Footnotes

1 The three excluded questions are: (1) Do you regularly participate in a hobby or leisure activity?, (2) Do you get friends or family around for a drink or meal at least once a month, and (3) Do you have two pairs of properly fitting shoes, including a pair of all weather shoes?

2 We present estimated marginal effects as opposed to odds ratios as Ipek [32] argues that it is incorrect to make a coefficient comparison based on the size of an odds ratio as it does not include information about the probability of the outcome variable.

## Code availability

The authors can provide the R code upon request.
